# Stakeholder perceptions of forest disturbance drivers and management responses are aligned in Central Europe

**DOI:** 10.1007/s13280-026-02397-9

**Published:** 2026-04-30

**Authors:** Michaela Perunová, Roman Modlinger, Vilém Jarský, Tomáš Hlásny

**Affiliations:** Faculty of Forestry and Wood Sciences, Czech University of Life Sciences in Prague, Kamýcká 129, 165 00 Prague 6 –Suchdol, Czech Republic

**Keywords:** Climate-change adaptation, Forest disturbance perception, Questionnaire-based survey, Social-ecological systems, Value-action gap

## Abstract

Forest disturbances are increasingly understood as social-ecological phenomena involving diverse actors. Here, we focused on one of the Europe’s largest recent disturbances—the drought-triggered bark beetle outbreak in Central Europe (2017–2022). Based on a survey of 165 respondents, we examined perceived disturbance drivers, response actions, and the alignment between them as an indicator of a value-action gap that is often present in social-ecological and governance systems. Climatic and forest structure-related factors were identified as the dominant drivers, aligning with current scientific understanding, whereas socio-economic and management constraints were perceived as less influential. Reported responses reflected awareness of climate-change risks and involved workforce training, adaptive change in species composition, and water-retention measures. Cause–action alignment was observed in nearly half of respondents, particularly among managers and legal entities. We found a relatively sound understanding of disturbance causes and corresponding responses, likely shaped by recent experience with an unprecedented disturbance impact.

**Supplementary Information:**

The online version contains supplementary material available at https://doi.org/10.1007/s13280-026-02397-9.

## Introduction

Forest disturbances are not merely ecological events, but also socially constructed phenomena, shaped, interpreted, and acted upon through individual cognition, institutional frameworks, and cultural context (Messier et al. 2013; Folke et al. 2016). Understanding how various actors perceive and respond to forest disturbances is essential for effective governance but also for enhancing social-ecological resilience, i.e., the ability of human–nature systems to adapt to or transform in response to changes, while maintaining functions that support human well-being (Biggs et al. 2021). The importance of such an integrated social-ecological context is underlined by the frequent misinterpretations and conflicts, which surround disturbance causes and management responses and vary across stakeholders (Blicharska and Angelstam 2010; Mack et al. 2023), local communities and organizations (Prentice et al. 2018). The importance of a coupled social-ecological context has increased with the recent disturbance intensification in Europe (Hlásny et al. 2021a; Patacca et al. 2023; Hlásny et al. 2025a). The major disturbance pulse after 2018, triggered by extreme climatic conditions—likely the largest in Europe in at least the past 170 years (Senf and Seidl 2021)—highlighted the complex multi-actor nature of novel disturbance dynamics (Hlásny et al. 2021a, b).

Here, we used the Czech Republic (Central Europe) as a case study, as it was one of the regions in Europe that were most severely affected by the recent disturbance wave (Hlásny et al. 2021b; Washaya et al. 2024). The millennial drought of 2018 triggered a large-scale outbreak of spruce bark beetle (*Ips typographus*), which affected between 2017 and 2022 approximately 100 million cubic metres of forest growing stock and led to the clearing of several hundred thousand hectares of forest (Washaya et al. 2024, 2025). The extent and severity of this event have challenged several traditional paradigms and long-held beliefs in Central European forestry, such as the sustainability of spruce monoculture management (Hlásny et al. 2021a, b; Knutzen et al. 2025), the misinterpretation of nature-conservation areas as bark beetle sources (Montano et al. 2016; Krüger et al. 2025), and the underestimation of climate-change risks combined with an overreliance on active outbreak control methods like sanitation logging, mass trapping, and insecticide use (Hlásny et al. 2021a). Moreover, large-scale bark beetle outbreaks in Europe have been historically surrounded by controversies about disturbance causes and impacts as well as management actions and their collateral effects, regularly causing public upheavals and even political conflicts (Müller 2011; Blicharska and Smithers 2018).

Here, we used the perspective gained three years after the disturbance to survey forestry stakeholders on the perceived causes of the outbreak—ranging from lacking workforce to climate change—and the actions taken in response, considering respondents’ work position (e.g., manager, employee) and ownership type (e.g., state, private). Building on these insights, our next objective was to assess how the reported actions align with these perceived causes across stakeholder types and whether certain stakeholder groups show greater consistency between their conviction about outbreak causes and their corresponding actions than others.

We hypothesize that respondents will assign high importance to management, logistical, and labor-related constraints, which feature prominently in public discourse, while climate change and the high share of spruce will be rated less prominently, contrary to prevailing scientific evidence (Hlásny et al. 2021a). This would reflect the historical economic importance of spruce in regional forestry (Knoke et al. 2021), limited availability of economically viable alternatives, and insufficient awareness of climate-related risks. We further hypothesize that alignment between perceived causes and reported management responses will vary among stakeholder groups—defined by professional role and ownership type—reflecting the interplay between increasing exposure to climate-change education and the constraining effects of rigid legislation, economic pressures, and cultural, practical, and institutional barriers.

## Data and methods

### Study area and studied disturbance event

Forests of the Czech Republic (Central Europe) cover 34.2% of the country (2.69 mil. ha). The forestry sector contributes less than 0.4% to the national Gross Value Added (GVA) and employs approximately 13 500 people. Downstream sectors, including the wood-processing industry, the pulp and paper industry, and furniture manufacturing, contribute an additional 1.08% of GVA to the national economy. Forests are predominantly state-owned (53.7%), followed by private owners (19%), municipalities and cooperatives (17.1%), and churches and religious organizations (5.4%). There are around 411 000 forest owners in total in the country (Ministry of the Agriculture of the Czech Republic 2025).

The studied disturbance event was the post-2018 outbreak of spruce bark beetle *I. typographus*, unprecedented in recorded history. Between 2017 and 2022, approximately 100 million m^3^ of wood was affected by bark beetles and salvaged, with 32 million m^3^ removed in the peak outbreak year of 2021. For comparison, the pre-disturbance average annual planned harvest level was around 15 million m^3^. The core outbreak area spanned 9000 km^2^ (11% of the country’s forests) and lost 38% of its forest cover (Washaya et al. 2024). The area comprised properties of 172 319 forest owners, predominantly under state ownership (38%), followed by natural persons (30.5%) and municipal forests (22%). The scale of this event required active state intervention, including subsidy and compensation schemes, the adoption of country-wide zonation with differentiated management priorities, and other emergency measures.

### Questionnaire design

We investigated respondents’ responses to seven questions organized in four categories: (i) property characteristics, (ii) respondent characteristics, (iii) perceived causes of bark beetle outbreak, and (iv) measures taken in response to the outbreak. The questionnaire included a combination of multiple-choice and scaled-response items. Scaled questions were rated on a Likert-type scale ranging from 0 to 5, where 0 represented the smallest perceived effect on the outbreak or the smallest conviction about the extent of response action implemented, and vice versa.

**Fig. 1:**
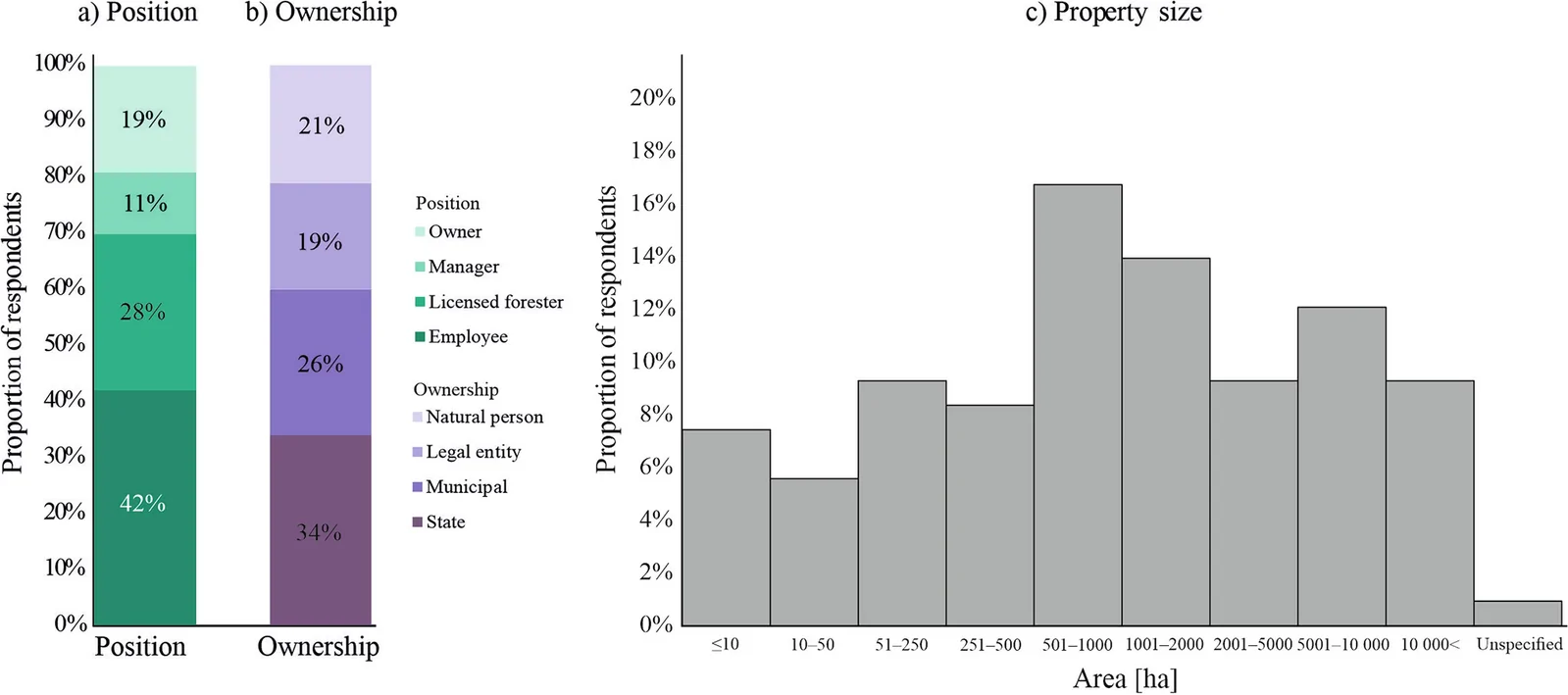
Respondent proportions with regard to work-position type (**a**), forest ownership type (**b**), and property size (**c**)

Respondents represented a broad range of forest stakeholders, spanning different professional roles, ownership types, and property sizes (Fig. 1, Appendix A). The considered working positions were employees (42%), licensed foresters (professionals providing management supervision for different owners; 28%), forest owners (19%), and forest managers (11%) (Fig. 1a). To facilitate the analyses, the reported ownership types were merged into four categories: natural person (21%), legal entities (19%), municipal forests (26%), and state forests (34%) (Fig. 1b) (Jarský et al. 2023). In terms of the property size, the distribution was relatively balanced, with the largest share of respondents belonging to the category of 500–1000-hectare properties (18%) (Fig. 1c). The respondents included 94% males, 4% females, and 2% individuals who did not specify their gender.

The printed questionnaire was distributed through two workshops and individual interactions with respondents, while the digital questionnaire was distributed through forest owner associations, major forest enterprises, and individual contacts of the author team. The data collection was carried out from February to June 2024, resulting in the completion of 165 questionnaires.

To obtain responses on perceived outbreak causes, the respondents were asked:

*“Which of the following factors do you expect to have had the strongest effect on (i) triggering the outbreak and (ii) its development?”*

For corresponding response action, the question was:

*“Which measures (i) did you implement in response to the outbreak, or (ii) do you plan to implement in the foreseeable future?”*

Since each respondent gave two answers for each question, we used the maximum of the two values in the statistical analysis. The full list of response options for the causes and actions is provided in Tables 1 and 2, respectively.

**Table 1:** Categories of questions on the causes of bark beetle outbreak as perceived by the respondents and specific causes addressed by the questions. Abbreviations used as labels in graphs are indicated

Category	Abbrev	Outbreak causes	Abbrev
Forest-related factors	Forest	High spruce proportion and low species diversity	Spruce
		High forest age	Forest age
		High spruce growing stock	Growing stock
Climate-related factors	Climate	Climate change as a complex phenomenon	Climate change
		Extremely hot a dry period 2015–2018	Drought
Socio-economic factors	SocEco	Insufficient workforce	Workforce
		Management restrictions related to the COVID crises	COVID
		Reduced timber price with adverse impact on forestry operations	Timber price
Forestry legislation	Legislation	Management restrictions due to nature conservation	Nature conservation
		Inefficient procurement of harvesting operations	Procurement
Disturbance management	Management	Insufficient use of pheromone traps and trap trees	Trapping
		Low intensity of debarking and chemical treatment of infested logs	Log treatment
		Bark beetle spread through the transportation of infested logs	Log transport
		Insufficient salvage logging operations	Salvaging

**Table 2:** Categories of questions on the actions taken in response to the outbreak by the respondents and specific actions addressed by the questions. Abbreviations used as labels in graphs are indicated

Category	Abbrev	Actions taken	Abbrev
Forest vulnerability-related response actions	Forest	Reduced use of spruce in regeneration	Spruce
		Active game control to protect forest regeneration and maintain species diversity	Game
		Reduced use of clear-cutting management	ClearCut
		Reduced forest rotation period resulting in reduced forest age	Rotation
Climate-related response actions	Climate	Application of water-retention measures (ponds, stream regulation, etc.)	Water
		Prayers for water	Prayer
Responses related to socio-economic condition actions	SocEco	Employee education	Education
		Increase in workforce	Workforce
		Financial buffers for overcoming calamity periods	Buffer
		Economic activity diversification	Diverse
Disturbance management-related response actions	Management	Increased frequency of preventative inspections aimed at identifying bark beetle infestations	Inspections
		Increased used of measures such as mass trapping of beetles and sanitary harvesting of infested trees	Control
		Increase in harvesting capacity to apply the early removal of infested trees more efficiently	Harvest

The perceived causes of the outbreak were grouped into five categories (see Table 1 for specific response options):

- **Forest-related factors** focused on conditions that heightened forests’ susceptibility to bark beetle infestations, typically resulting from past management (e.g., high proportion of Norway spruce, high forest age and tree density, etc.).
- **Climate-related factors** included one option attributing the outbreak to the singular exceptionally dry period from 2015 to 2018, without explicitly linking it to climate change, and another attributing the outbreak to climate change as a broader phenomenon.
- **Socio-economic factors** addressed the fact that the timing of the outbreak coincided with (i) the COVID-19 crisis and related restrictions on management operations, and (ii) a substantial drop in timber prices, reducing owners’ possibilities to harvest infested trees.
- **Forestry-legislation factors** included (i) management restrictions under nature-conservation regulations, which are often cited by foresters as contributing to bark beetle outbreaks in Europe; and (ii) a Czech-specific rule requiring state forest enterprises to publicly tender harvesting, including sanitation felling, which can delay early removal of infested trees and reduce control effectiveness.
- **Deficiencies in outbreak-management practices** included insufficiently applied mass trapping of bark beetles, insufficient and/or delayed removal of infested trees, low intensity and frequency of debarking and chemical treatment of infested logs, and the possible expansion of the outbreak via transportation of untreated logs.

The response actions reportedly taken by the respondents represented the following four categories (see Table 2 for specific response options):

- **Forest vulnerability-related responses** included measures aimed at reducing forest vulnerability by lowering the proportion of spruce in the regenerating forest cohort, shortening forest rotation periods (thus reducing forest age), controlling game to increase the diversity of the regenerating forest cohort, and avoiding clear-cutting management that leads to a simplified and vulnerable forest structure.
- **Climate-related responses** involved measures aimed at water retention in forests through small-scale structures such as ponds, as well as stream and road regulation, and prayers for water, which have been practiced in, at least, some church-owned forests.
- **Responses related to socio-economic conditions** encompassed measures aimed at increasing the workforce and improving education, creating financial buffers, and diversifying financial portfolios to increase stability under future disturbance impacts.
- **Outbreak management-related responses** included measures aimed at increasing capacity for early removal of infested trees, increasing the frequency of regular forest inspections to identify infestation foci, and expanding the use of control measures focused on mass trapping of beetles.

### Statistical analysis

Generalized linear mixed effect models (GLMM) were used to evaluate the perceived causes of and responses to the disturbance across different ownerships and positions. The main cause and response categories (Tables 1 and 2), ownership type and working position were used as fixed effects, while respondent was defined as a random effect. The response variable was an integer between 0 and 5, representing the degree of conviction about the cause and response. To apply the beta regression model, we transformed the response values to fall strictly within the (0, 1) interval using the adjustment proposed by Cribari-Neto and Zeileis (2010):

$$\left( {Y \times \left( {N - 1} \right) + 0.5} \right)/N,$$

where Y is the response variable and N is the sample size. Model selection was performed using forward selection, with model quality evaluated using the Akaike Information Criterion (AIC). The significance of individual predictors and their interactions was assessed using likelihood ratio tests implemented via the *drop1* function (Zuur et al. 2009). Pairwise comparisons among significant predictors and interactions in the final model were conducted using Tukey-adjusted contrasts with the *emmeans* package (Lenth and Piaskowski 2025). The R package (v. 4.4.2.; R Core Team 2024) glmmTMB was used following the procedures proposed by Brooks et al. (2017).

**Fig. 2:**
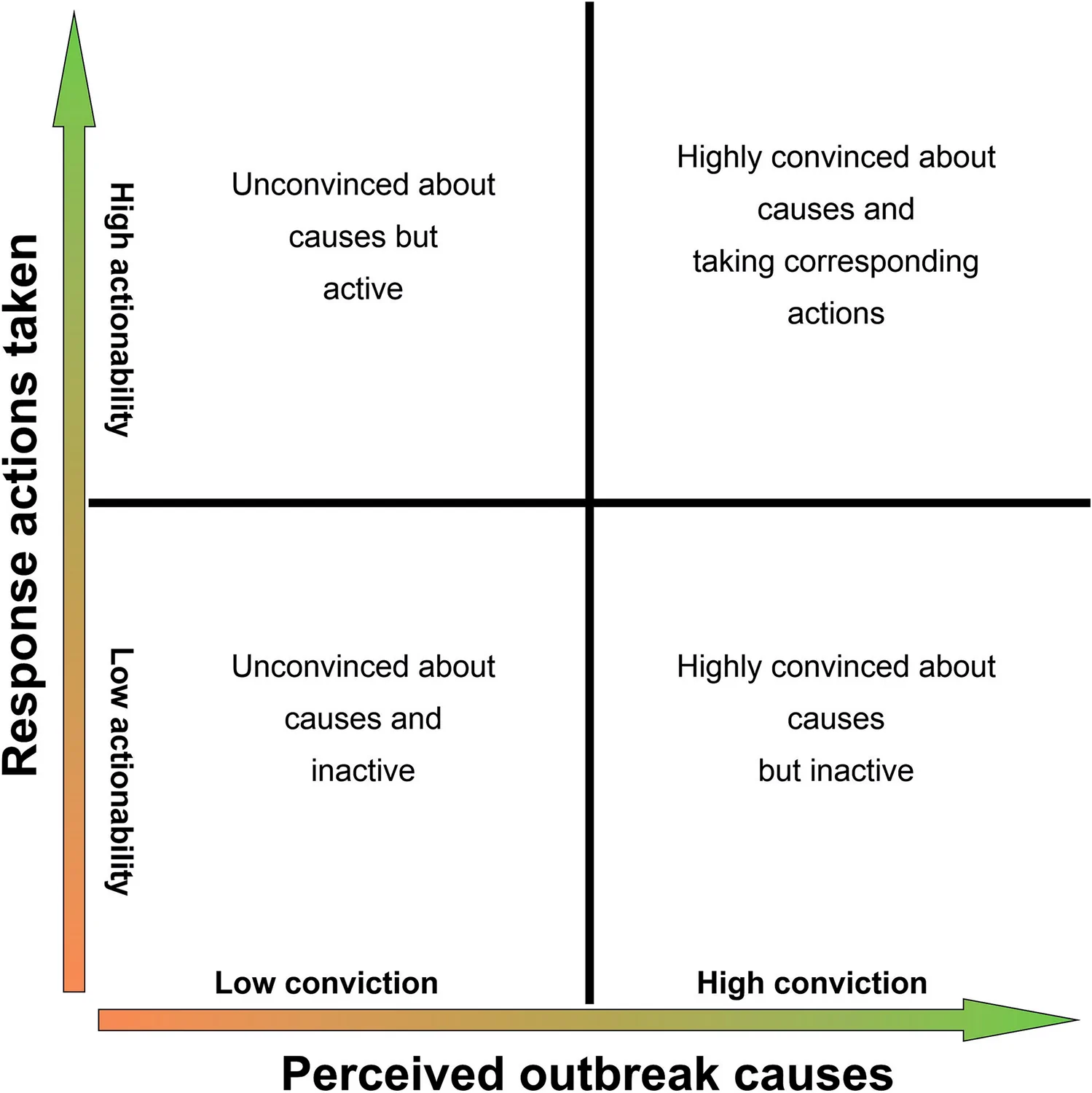
Diagram illustrating the evaluation of alignment between management actions taken in response to the disturbance and the corresponding perceived causes

To examine the alignment between stakeholders’ perceptions of outbreak causes and their reported response actions, we selected a subset of response options from Tables 1 and 2 and constructed 16 logical pairs (Table 3, Appendix B). We note that multiple response actions could be associated with a single cause, and conversely, the same action could be considered a response to more than one perceived cause. The overall distribution of cause-response pairs was partitioned into four quadrants within a two-dimensional space defined by the 0–5 scales of perceived causes (*X*-axis) and response actions (*Y*-axis). The typology of respondents determined by their position in quadrants is indicated in Fig. 2. The analysis was subsequently stratified by the respondent’s position type and the ownership type.

**Table 3:** Combinations of perceived outbreak causes and corresponding response actions reported by respondents used to assess the alignment between disturbance perceptions and actions

Perceived outbreak cause	Abbrev	Action taken in response	Abbrev
High spruce proportion and low species diversity	Spruce	Reduced use of spruce in post-disturbance regeneration	Spruce
		Active game control to protect forest regeneration and maintain species diversity	Game
		Reduced use of clear-cutting management	ClearCut
High forest age	Forest age	Reduced forest rotation period resulting in reduced forest age	Rotation
Climate change as a complex phenomenon	Climate change	Employee education	Education
		Reduced use of spruce in post-disturbance regeneration	Spruce
		Application of water-retention measures (ponds, stream regulation, etc.)	Water
Insufficient workforce	Workforce	Increase in workforce	Workforce
Management restrictions related to the COVID crises	COVID	Financial buffers for overcoming calamity periods	Buffer
Reduced timber price with adverse impact on forestry operations	Timber price	Financial buffers for overcoming calamity periods	Buffer
		Economic activity diversification	Diverse
Insufficient use of pheromone traps and trap trees	Trapping	Increased use of measures such as mass trapping of beetles and sanitary harvesting of infested trees	Control
		Increased frequency of preventative inspections aimed at identifying bark beetle infestations	Inspections
Insufficient salvage logging operations	Salvaging	Increase in harvesting capacity to apply the early removal of infested trees more efficiently	Harvest
Low intensity of debarking and chemical treatment of infested logs	Log treatment	Increase in workforce	Workforce

## Results

### Model summary

The beta regression model fitted to the data on perceived disturbance causes yielded an *R*^2^ of 0.68 (marginal *R*^2^ = 0.46). The only significant predictor was the category of causes (Table 1), while working position, ownership type, and their interaction were not significant at *α* = 0.05. For the model of response actions, the *R*^2^ was 0.59 (marginal *R*^2^ = 0.48). In this case, the response action category (Table 2), working position, and ownership type were all significant predictors and were used for interpretation. No significant interaction among factors was identified.

#### Perceived disturbance causes

Climate-related factors received significantly higher scores than all the remaining categories (fitted value 3.67 on the 0–5 scale) and were significantly different from all remaining factors (contrast *t*-test; *p* < 0.001; Appendix C). Conversely, socio-economic factors received the lowest scores of 2.52 and differed significantly from all other categories (*p* < 0.001). No significant differences were observed between forest-related factors (3.07), disturbance management (3.06), and forestry legislation (3.08).

A closer look at individual responses (Table 1) showed that, within the climatic category, respondents ranked “extremely hot and dry weather during 2015–2018” (4.0) significantly higher than “climate change as a complex phenomenon” (3.6; *p* < 0.001; Appendix C). Among forest-related factors, a high proportion of spruce received the highest rating (3.6) and differed significantly from high stand age (2.8; *p* < 0.001) and high growing stock (3.0; *p* < 0.01), whereas high growing stocks and high age were perceived similarly (*p* = 0.31). Within the socio-economic category, COVID-related restrictions were perceived as relatively uninfluential (1.1), while management constraints associated with labor shortages and reduced timber prices received similarly high scores 3.3 and 3.4. In the legislative category, nature-conservation-related management restrictions received relatively low scores (2.7), whereas inefficient procurement procedures were rated considerably higher (3.5; *p* < 0.001).

Within the disturbance management category, “insufficient use of pheromone traps and trap trees” received comparatively low importance (mean score 1.8; *p* < 0.001). In contrast, “insufficient salvage logging operations” was rated as the most prominent management-related cause (3.7). Other factors, including “bark beetle spread through the transport of infested logs” and “low intensity of debarking and chemical treatment,” were also perceived as influential, with scores of 3.3 and 3.4, respectively.

#### Management responses

Forest-related response actions were rated significantly higher (fitted value 3.16; contrast *t*-test *p* < 0.001) compared to all the remaining response categories, i.e., climate-related (2.61), outbreak management (2.33), and socio-economic (2.37). At the same time, climate-related factors received significantly higher scores than outbreak management and socio-economic factors (*p* < 0.05), though the difference in fitted values was not so distinct.

**Fig. 3:**
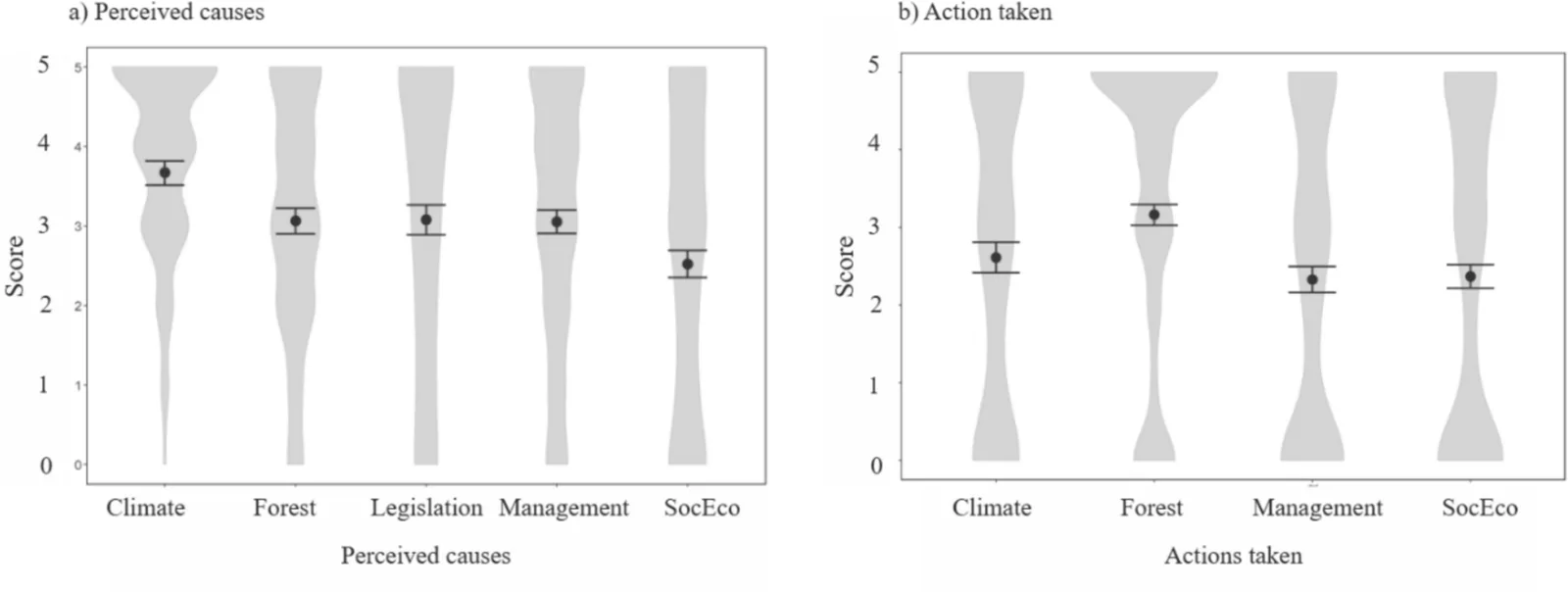
Perceived causes of the outbreak (**a**) and management actions taken (**b**) for the main factor categories, estimated using GLMMs. Dots represent fitted values, whiskers the 95% confidence intervals, and violins the distribution of the original values

Respondents ranked forest-related responses highest across all work position and ownership types, although with varying significance, with no group deviating from the general pattern shown in Fig. 3. Substantial variation was observed in climate-related actions: these were rated particularly highly by managers (3.09) but received lower scores among forest owners (2.24) and natural persons (2.06). Unlike other work positions, forest managers attributed as much importance to climate-related response actions as to forest-related actions. No distinct patterns were identified for the remaining combinations of work position and ownership type.

Within the category of climate-related actions, water-retention measures received a moderate rating (3.27), whereas prayers for water were ranked significantly lower (1.99; *p* < 0.001). Within the forest-related category, reducing the use of spruce in regeneration received the highest score (3.76), while shortening rotation age was rated lowest (1.98) and was significantly different from all the other forest-related actions (*p* < 0.001). Increased game control and reduced reliance on clear-cutting were rated similarly, with fitted values of 3.51 and 3.62.

**Fig. 4:**
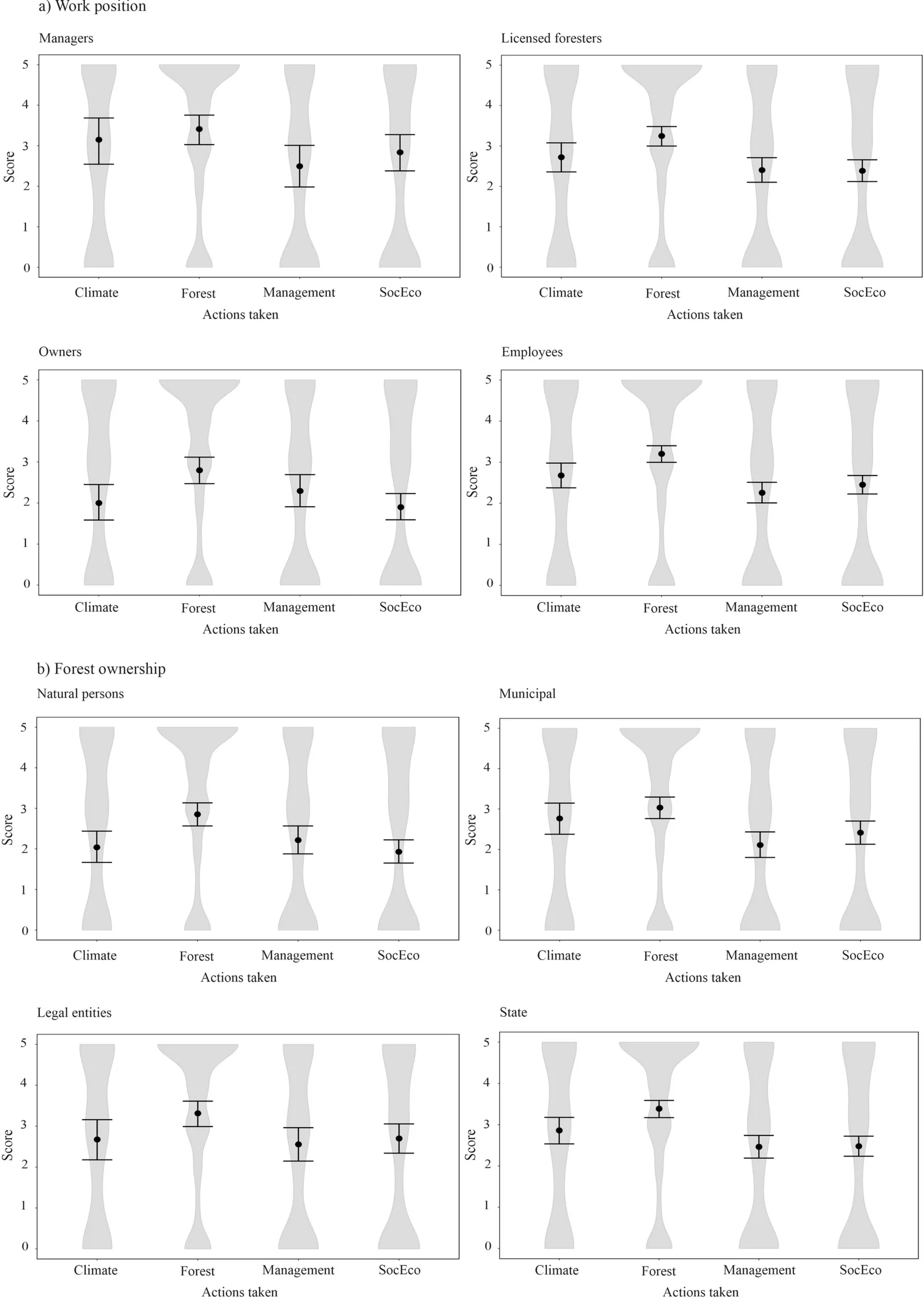
Differences in actions reportedly taken across respondent work-position types (**a**) and forest ownership types (**b**). Dots represent fitted values, whiskers the 95% confidence intervals, and violins the distribution of the original values

Considerable variation was observed within the socio-economic category (Fig. 4). Measures aimed at an increased workforce and diversified economic activities received the lowest scores (1.6 and 1.78, respectively). Establishment of financial buffers received moderate importance (2.88), while the highest importance was attributed to employee education (3.34). Within the disturbance management category, increasing the frequency of preventive forest inspections received the highest rating (2.34), whereas the remaining actions—increased use of beetle trapping and sanitary logging of infested trees, and expansion of salvaging capacity—were rated lower (fitted values 3.42 and 1.4, respectively).

### Alignment of causes and responses

**Fig. 5:**
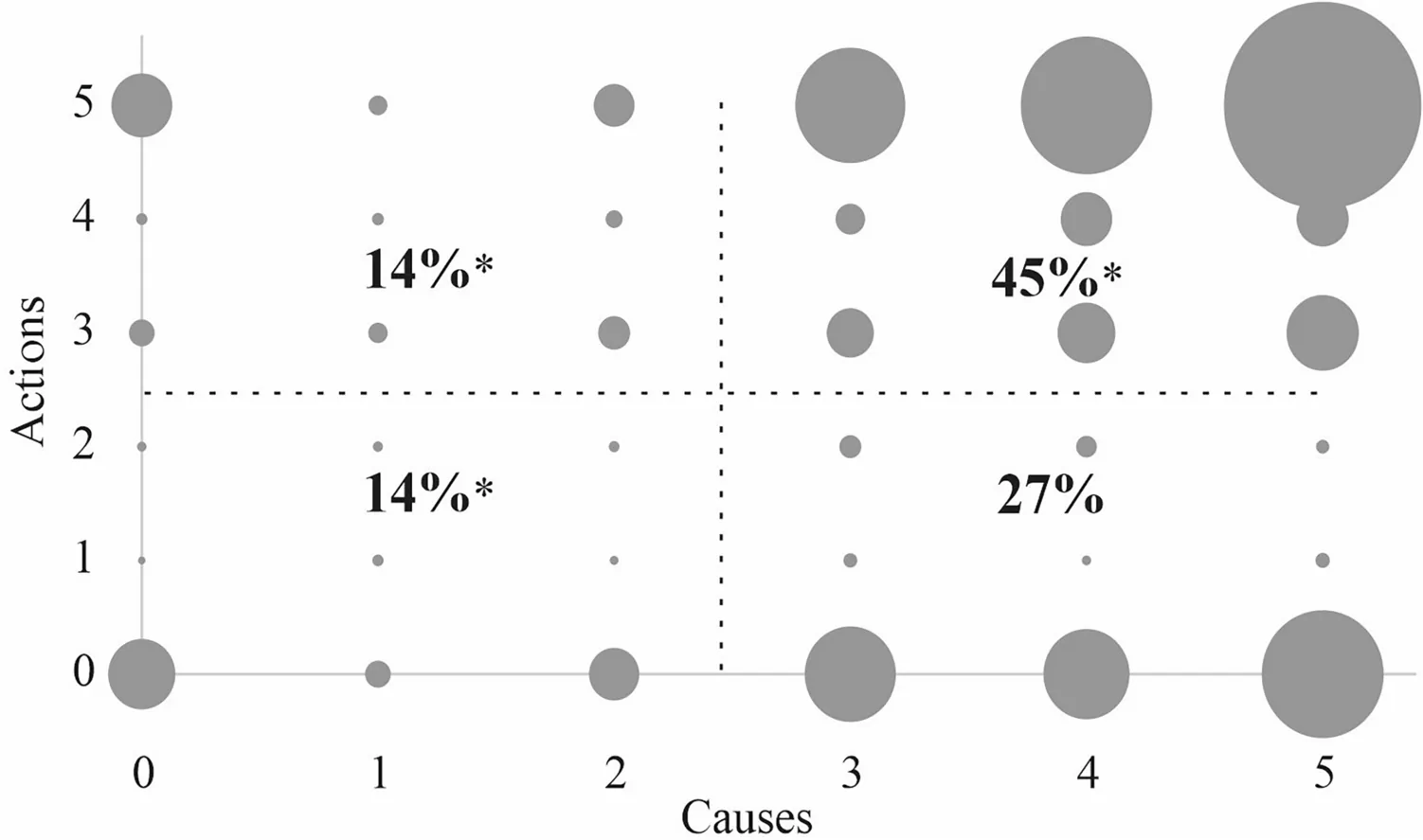
Relationship between perceived outbreak causes and actions taken among all respondents. The horizontal axis (causes) represents the degree to which respondents perceived specific causes as relevant, while the vertical axis (actions) reflects the extent to which they reported implementing specific response measures. Each data point was sized proportionally to the number of cases within each cause-action combination. Relative proportions of cases are indicated for each quadrant. An asterisk denotes a statistically significant deviation from an even distribution (i.e., 25% of responses in each quadrant) at *p* < 0.05

Among all respondents, 45% of responses (*n* = 2640; 165 respondents × 16 question pairs, Table 3) showed alignment between the perceived causes and the reported response actions (Fig. 5), falling into the top-right quadrant (see scheme in Fig. 2). In contrast, 27% of responses were in the bottom-right quadrant, representing strong opinions about the causes that are not followed with corresponding actions. The top-left quadrant (“low perception–high action”) contained 14% of responses, indicating actions taken despite weakly perceived related causes. Lastly, 14% of the respondents indicated a lack of awareness combined with inaction.

**Fig. 6:**
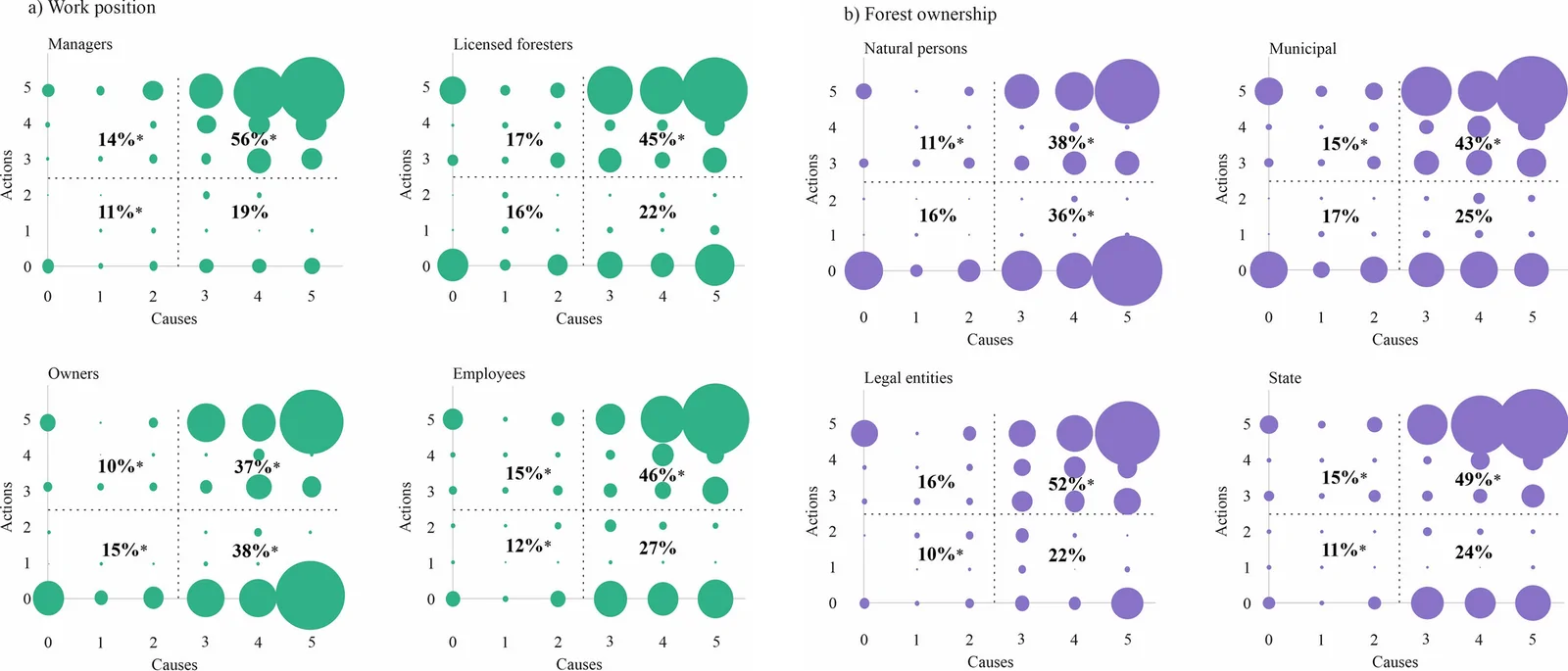
Distribution of perceived causes of the bark beetle outbreak and the actions taken by work position (**a**) and forest ownership (**b**) type categorized into quadrants

At the level of work position (Fig. 6), managers showed the highest alignment (56%) between perceived causes and reported responses. At the same time, 19% reported strong conviction regarding the causes but did not take related action. A lack of awareness combined with inaction was identified in 11% of managers. Finally, 14% provided a difficult-to-interpret response pattern, reporting that actions were taken despite limited perception of the underlying causes.

Forest owners appeared at the opposite end of the spectrum. Only 37% indicated that awareness of causes was followed by action, while 38% recognized the causes but did not respond. Low awareness combined with inaction was reported by 15%, and low awareness followed by action was reported by 10% of respondents. The remaining position types—licensed foresters and employees—ranked between these two groups.

Among all ownership types, legal entities showed the strongest alignment between perceived causes and responses (52%). In addition, 22% of responses indicated recognition of causes without follow-up action. A further 10% reported low awareness combined with inaction, while 16% indicated that actions were taken despite only a weak perception of the causes. The response pattern of state forest owners was nearly identical to that of legal entities, although the top-right quadrant (cause-action alignment) accounted for only 49% of responses.

The lowest level of alignment was found among natural persons (38%), while 36% of responses showed recognition of causes without subsequent action—the highest proportion of this category across all groups. Another 16% reflected low engagement in both domains, and 11% reported taking action without strong conviction regarding the causes.

A moderate level of alignment was observed among respondents representing municipal forests, with 43% of responses indicating consistent cause recognition and action. A further 25% indicated that although causes were perceived, no corresponding action followed. Finally, 15% of responses reflected action taken without strong perception of causes, and 17% reported low awareness coupled with inaction.

## Discussion

Here, we provided novel insights into perceptions of, and responses to, a disturbance event that exceeded all previously recorded events in the history of organized forest management in Central Europe. The obtained picture contradicts some earlier assessments (e.g., Sousa-Silva et al. 2018) as well as our hypotheses: we identified a sound understanding of disturbance causes, followed by a substantial share of respondents acting in line with this understanding. Nevertheless, we also identified considerable variation among stakeholder groups, with institutional actors exhibiting higher cause-action alignment than other categories. These findings can inform strategies for overcoming barriers to science-based management responses and for translating recent disturbance experience into better coordinated actions.

### Perception of disturbance causes

Strong control over forest development is deeply rooted in Central European forestry tradition (Messier et al. 2019; Stritih et al. 2026), generally directing attention toward disturbance causes that can be addressed through management and for which responsible actors can be identified. Recognizing the prominence of climatic factors as the cause of recent unprecedented disturbance is therefore surprising and indicates that the respondents acknowledge (i) the importance of drivers that lie outside the scope of operational control, but also (ii) the fact that the forests were not adapted to accelerating climate change, admitting underestimation of emerging risks in recent decades. Acknowledging the importance of climatic factors also aligns with the prevalent scientific understanding of the role of climate change in disturbance intensification in Europe (Seidl et al. 2011, 2017; Grünig et al. 2026). Such an outcome corresponds to the previously identified increasing awareness of climate-change-related risks among foresters that has been reported in various social-ecological contexts (Sousa-Silva et al. 2018; Himes et al. 2023). Although these findings are encouraging, respondents were leaning slightly more to an isolated extreme drought event than to climate change as a broader phenomenon. This distinction may have practical implications, as isolated episodes may not require systemic management changes. However, the close conceptual overlap between the two factors may have obscured this distinction for respondents.

Even though the forest species and structural homogeneity are other prominent drivers of disturbance susceptibility with strong scientific support (Seidl et al. 2011), forest-related factors were not perceived as the prominent cause, attaining the same score as legislative and outbreak-management factors. Nevertheless, the average score of 3.16 assigned to forest-related factors—and particularly the high rating of 3.9 for the prevalence of commercially important yet vulnerable spruce—indicates that these factors are well recognized, even as foresters contend with the lack of commercially viable alternatives and the potentially high costs of transitioning to other management systems (Stritih et al. 2026).

Although the socio-economic category received a relatively low score of 2.52, this was due to the minor importance of COVID-related restrictions, a category that may have been too abstract for the respondents. The other two factors—labor shortages (possibly partly related to the COVID crisis) and the timber price decrease coinciding with the disturbance peak (Asada et al. 2023)—were ranked relatively high (3.43 each). Overall, socio-economic factors should still be considered important despite their lower average score.

We found no significant effects of work positions and ownership types. This is unexpected, given the likely variation in education and training between forest managers and employees as well as the different responsibilities for the outcome of forestry activities (e.g., managers and owners vs. employees). However, this outcome can be related to the complex design of the statistical analysis and the limited number of respondents in some categories.

### Pattern in the reported response actions

Regarding actions reportedly taken in response to the disturbance, measures aimed at reducing forest vulnerability received the highest scores. The highest-rated measure—reducing the proportion of spruce in regeneration—reflects awareness of the risks associated with its use; however, the effects of this measure will materialize only in the long term. In contrast, measures that could deliver more immediate benefits, such as shortening rotation periods (Zimová et al. 2020) and reducing the use of clear-cutting management (Messier et al. 2013), received lower scores. This pattern may suggest that though the risks are recognized, their urgency may not yet be fully acknowledged. Although climate-related disturbance causes had a high recognition, the related response actions received a moderate rating. This can be due to specific response actions included in this category (water-retention measures and prayers for water), which do not belong to the typical portfolio of management operations in Central Europe. Among socio-economic response actions, measures aimed at enhancing socio-economic resilience to future shocks, such as financial buffers (Hlásny et al. 2021a), received relatively low scores, likely reflecting limited experience and institutional tradition with such approaches. By contrast, employee education received high ratings, probably reflecting both its perceived relevance and its relatively easy implementation, supported by the broad availability of training programmes.

### Coherence of perception and action

The results showed the prevalent coherence between cause understanding and the corresponding actions. This is a more optimistic pattern than in the earlier assessment by Sousa-Silva et al. (2018), who found that while European foresters were broadly aware of climate-change risks, only about 10% reported taking actions. This finding suggests that while climate-change risks were previously seen mainly as future concerns, the 2018 disturbance wave made them immediate and salient, likely shifting stakeholders’ perceptions and willingness to act. However, the different types of response actions addressed in our research may limit the comparability. Further, 27% of respondents recognized the causes but did not take corresponding actions. Obviously, cognitive, practical, and structural barriers can still prevent them from translating awareness into actions (Kollmuss and Agyeman 2002; Hengst-Ehrhart 2019). Nonetheless, the large proportion of respondents in this category can still be seen as a positive outcome, since cause understanding positively shapes decision-making and influences how individuals engage in adaptation measures (Blennow et al. 2012; Yousefpour and Hanewinkel 2015; van Valkengoed et al. 2022). The latter two respondent groups can be particularly suitable targets for communication and training programmes (Brink and Wamsler 2019; Vulturius et al. 2020; Nikinmaa et al. 2024; Fouqueray et al. 2026). In contrast, the two remaining groups (active without understanding, and unaware and inactive) may be harder to engage. Since they represent 28% of the total sample, their motivations would deserve further research.

A closer look into the ownership and work position profiles indicates that forest managers, state and legal entities (e.g., private forest enterprises) show the highest cause-action alignment. This may reflect both their formal responsibilities and their embeddedness within institutional logics that prioritize regulation, stewardship, and accountability (Müller 2011). In contrast, forest owners and natural persons—mostly representing small holdings—exhibited a larger discrepancy between problem recognition and intervention. The reasons are likely multifaceted: These stakeholders often manage forests alongside other occupations, and may lack the time, financial resources, or technical capacity to understand disturbance causes and implement corresponding measures. In Central Europe, small owners are often culturally and professionally disconnected from their forests, a legacy of post-socialist land restitution after 1990, which returned ownership to many individuals after decades of state management (Jarský et al. 2018). Moreover, small-holding size may constrain responses to large-scale disturbances, reflecting the frequent institutional-ecological mismatch (Ekstrom and Young 2009). Collectively, these factors suggest that these stakeholder groups may require tailored approaches, including advisory services and training, as instruments designed for large institutional owners may not be effective here.

While the presented results offered several novel insights, they also open new questions on specific motivations and barriers of different stakeholders to act, such as lack of time and finance, access to informational, technological, and human resources (Yousefpour et al. 2017; Fischer et al. 2022; Roitsch et al. 2023), or a scale mismatch between institutions and ecological problems (Ekstrom and Young 2009). These findings suggest that a better understanding of the specific challenges and motivations faced by different stakeholders is essential for designing measures and policies that are practical and effective on the ground.

## Supplementary Information

Below is the link to the electronic supplementary material.

Supplementary file1 (PDF 712 kb)

## Data Availability

It is available from the corresponding author upon a reasonable request that does not contradict the Privacy Notice for the Survey.
