# Supplementary material for: Stakeholder perceptions of forest disturbance drivers and management responses are aligned in Central Europe
Source: Ambio. 2026 Apr 30;55(11):2691–704. doi: 10.1007/s13280-026-02397-9 (PMC13661826; doi:10.1007/s13280-026-02397-9)
Supplement: Supplementary file 1 [file 13280_2026_2397_MOESM1_ESM.pdf]

APPENDICES

Stakeholder perceptions of forest disturbance drivers and management responses are aligned in Central Europe

Michaela Perunová[1], Roman Modlinger[1], Vilém Jarský[1], Tomáš Hlásny[1*]

[1]Czech University of Life Sciences in Prague, Faculty of Forestry and Wood Sciences, Kamýcká 129, 165 00, Prague 6 –Suchdol, Czech Republic.

[*]corresponding author, hlasny@fld.czu.cz

Appendices

Appendix A. Characterization of respondents

Category	Characteristics	Absolute frequency	Relative frequency
**Ownership**	Natural person	39	21%
	Legal entity	26	19%
	State	57	34%
	Municipal	43	26%
**Position**	Owner	31	19%
	Manager	18	11%
	Forest management specialist	47	28%
	Employee	69	42%
**Property size (hectares)**	≤ 10	13	8%
	> 10 and ≤ 50	10	6%
	> 50 and ≤ 250	16	10%
	> 250 and ≤ 500	15	9%
	> 500 and ≤ 1000	30	18%
	> 1000 and ≤ 2000	25	15%
	> 2000 and ≤ 5000	16	10%
	> 5000 and ≤ 10000	22	13%
	> 10000	16	10%
	Unspecified	2	1%

Appendix B. Distribution of respondents by position and ownership type with corresponding cause-action pair counts

Type of respondents		Number of respondents at position	Number of cause-action pairs	n*
**Position**	Managers	18	16	288
	Forest management specialists	47	16	752
	Owners	31	16	496
	Employees	69	16	1104
**Ownership**	Natural persons	39	16	624
	Municipal	43	16	688
	Legal entities	26	16	416
	State	57	16	912

n = number of respondents at position x number of cause-action pairs*

Appendix C.

Fitted values of cause-actions by Categories and Questions.

Category	Questions	Fitted value
Climate-related factors	Climate change	3.55
Climate-related factors	Drought	4.01
Forest-related factors	Forest age	2.78
Forest-related factors	Growing stock	3.03
Forest-related factors	Spruce	3.57
Forestry legislation	Nature conservation	2.72
Forestry legislation	Procurement	3.50
Outbreak management	Log transport	3.30
Outbreak management	Log treatment	3.39
Outbreak management	Salvaging	3.73
Outbreak management	Trapping	1.83
Socio-economic factors	COVID	1.11
Socio-economic factors	Timber price	3.34
Socio-economic factors	Workforce	3.36

Pairwise contrast comparison of the questions regarding causes.

Category	Pairwise comparison of the questions	p-value
Climate-related factors	Climate change / Drought	0.000222347
Forest-related factors	Forest age / Growing stock	0.311911553
Forest-related factors	Forest age / Spruce	3.30E-06
Forest-related factors	Growing stock / Spruce	0.001690543
Forestry legislation	Nature conservation / Procurement	2.52E-06
Outbreak management	Log transport / Log treatment	0.954810015
Outbreak management	Log transport / Salvaging	0.022649969
Outbreak management	Log transport / Trapping	4.46E-14
Outbreak management	Log treatment / Salvaging	0.089843501
Outbreak management	Log treatment / Trapping	3.06E-14

Outbreak management	Salvaging / Trapping	1.69E-14
Socio-economic factors	COVID / Timber price	0
Socio-economic factors	COVID / Workforce	0
Socio-economic factors	Timber price / Workforce	0.991244296

Fitted values of response-actions by Categories and Questions.

Category	Questions	Fitted value
Climate-related factors	Prayer	1.99
Climate-related factors	Water	3.27
Forest-related factors	ClearCut	3.62
Forest-related factors	Game	3.51
Forest-related factors	Rotation	1.98
Forest-related factors	Spruce	3.76
Outbreak management	Control	1.39
Outbreak management	Harvest	3.42
Outbreak management	Inspections	2.34
Socio-economic factors	Buffer	2.88
Socio-economic factors	Diverse	1.78
Socio-economic factors	Education	3.34
Socio-economic factors	Workforce	1.60

Pairwise contrast comparison of the questions regarding reactions.

Category	Pairwise comparison of the questions	p-value
Climate-related factors	Prayer / Water	0.00000
Forest-related factors	ClearCut / Game	0.87150
Forest-related factors	ClearCut / Rotation	0.00000
Forest-related factors	ClearCut / Spruce	0.74917
Forest-related factors	Game / Rotation	0.00000
Forest-related factors	Game / Spruce	0.29142
Forest-related factors	Rotation / Spruce	0.00000
Outbreak management	Control / Harvest	0.00000
Outbreak management	Control / Inspections	0.00000
Outbreak management	Harvest / Inspections	0.00000
Socio-economic factors	Buffer / Diverse	0.00000
Socio-economic factors	Buffer / Education	0.04115
Socio-economic factors	Buffer / Workforce	0.00000
Socio-economic factors	Diverse / Education	0.00000
Socio-economic factors	Diverse / Workforce	0.70660
Socio-economic factors	Education / Workforce	0.00000
